# Reinitiating ocrelizumab with a full 600 mg dose after extended treatment interruption: No increased risk of infusion-related reactions

**DOI:** 10.1177/20552173261449688

**Published:** 2026-05-15

**Authors:** Lisa G Schoof, Laura Hogenboom, Zoé LE van Kempen, Joep Killestein, Bob W van Oosten, Eva MM Strijbis

**Affiliations:** MS Center Amsterdam, Neurology, Vrije Universiteit Amsterdam, Amsterdam Neuroscience, 1209 Amsterdam UMC location VUmc, Amsterdam, The Netherlands

**Keywords:** Multiple sclerosis, ocrelizumab, infusion-related reactions

## Abstract

**Background:**

Infusion-related reactions (IRRs) are a common side-effect of ocrelizumab (OCR), with the highest risk occurring during treatment initiation. To minimize IRR risk, the first dose is split into two 300 mg infusions over two weeks. Subsequent doses are administered every six months as a 600 mg infusion. After treatment interruptions, some physicians restart with two separate 300 mg infusions due to concern about IRRs. This study evaluates IRR occurrence following a 600 mg infusion after extended treatment interruption.

**Methods:**

This retrospective cohort study included all multiple sclerosis (MS) patients who received at least one 600 mg OCR infusion between June 2018 and January 2024 at Amsterdam UMC. Infusions were categorized by interval duration: normal (6 months), moderate (7–9 months) and long (>9 months). Logistic regression analyzed the IRR occurrence.

**Results:**

1438 infusions, in 275 patients, were analyzed. Neither the moderate interval group (306 infusions, OR = 0.914, *p* = 0.77) nor the long interval group (56 infusions, OR = 0.928, *p* = 0.575) had significantly higher odds of developing IRRs compared to the normal interval group.

**Conclusion:**

Despite the retrospective design and smaller sample sizes in the moderate and long interval groups, our findings indicate that administering OCR as a single 600 mg infusion after a prolonged interval is safe regarding IRR occurrence.

## Key point

Administering OCR as a single 600 mg infusion after a prolonged interval is safe regarding IRR occurrence.

## Introduction

Ocrelizumab (OCR) is a highly effective treatment for multiple sclerosis (MS). Infusion-related reactions (IRRs) are the most common side effect of OCR treatment, with the highest risk occurring at the initiation of therapy.^1,2^ Several precautions are taken, in accordance with the on-label instructions for OCR, to reduce the risk of IRRs. These include the administration of premedication (e.g. methylprednisolone and antihistamine), a slow administration schedule of 2 to 3.5 h,^2,3^ and splitting the first dose into two 300 mg infusions administered over two weeks. Subsequent doses consist of a single 600 mg infusion given every six months.^4^ The interval between infusions can be extended for various reasons, for example, pregnancy, recurring infections, B-cell tailored dosing or patient-related factors. Since the risk of IRRs is highest at the first OCR infusions, it is plausible that patients who have an extended dosing interval for any of these reasons may have an increased risk of IRRs when reinitiating treatment. However, formal studies evaluating IRR risk after missed doses are currently lacking.^5^ After treatment interruptions, some physicians choose to restart with two separate 300 mg infusions due to concern about IRRs. However, evidence is lacking that this will lower the risk of IRRs. Likewise, there is no evidence in the current literature that administering OCR as a single 600 mg infusion after a prolonged interval increases the likelihood of IRRs.^5^ Our objective was to evaluate the occurrence of IRRs after administration of a 600 mg infusion after extended interruption of treatment.

## Methods

This retrospective cohort study included all OCR infusions of MS patients who received at least one 600 mg OCR infusion in the Amsterdam UMC, location VUmc, between June 2018 and January 2024 and who gave informed consent to participate in the Amsterdam MS Cohort. Data was extracted on demographics, CD19+ B-cell counts, OCR treatment details and adverse events. Premedication included 100 mg methylprednisolone intravenously, 1000 mg paracetamol orally, 40 mg pantoprazole orally and an antihistamine. During this treatment period, the standard antihistamine premedication changed from clemastine 1 mg intravenously to levocetirizine 10 mg orally. From July 2019 onward, patients with no prior IRRs received 600 mg infusions in a 2-h schedule, instead of the 3.5 h administration schedule.^2,3^ From April 2020 to February 2021, B-cell extended dosing was standard due to the COVID-19 pandemic. IRRs were documented during infusion visits and at subsequent follow-up visits and were collected through medical record review. IRRs were defined as adverse events during administration of OCR or within 24 h afterwards, and The Common Terminology Criteria for Adverse Events (CTCAE) to classify their severity was used.^6^ The interval between two infusions was counted in days, rounded to months and categorized into three groups: ≤ six months (normal interval), 7 and 9 months (moderate extended interval) and ≥ nine months (long extended interval). Logistic GEE models with exchangeable correlation analyzed predictors of IRRs (infusion number, rate, premedication type, and CD19+ B-cell count) and the odds of IRRs across the three interval groups. Statistical analyses were conducted with SPSS Statistics software V.28.0 (IBM).

## Results

We included 275 patients with a cumulative total of 1988 infusions (300 mg infusions n = 550, 600 mg infusions n = 1438). The number of 600 mg infusions per patient ranged from 1 to 11 infusions (median: 5). Of all 600 mg infusions, 681 (47.3%) were given in a 3.5 h administration schedule, 632 (43.9%) in a 2 h administration schedule and for the remaining infusions (8.7%) the dosing schedule was unknown. Of all 600 mg infusions, 896 (62.3%) received clemastine, 538 (37.4%) levocetirizine and 4 (0.3%) no antihistamine at the patient’s preference (see Table 1).

**Table 1.** Infusion characteristics (600 mg).

	All 600 mg infusions n = 1438	Normal (6 months) n = 1076	Moderate (7–9 months) n = 306	Long (> 9 months) n = 56
Number of individual patients	275	265	165	47
Female (%)	174 (63.3%)	166 (62.6%)	108 (65.6%)	26 (46.4%)
Age at start OCR (mean, SD)	40.4 (10.8)	40.6 (10.9)	40.3 (10.7)	39.6 (10.4)
BMI (mean, SD)	24.5 (4.1)	24.6 (4.1)	24.5 (4.2)	23.5 (3.2)
MS type RRMS PPMS SPMS	230 (83.6%) 35 (12.7%) 10 (3.6%)	221 (83.4%) 35 (13.2%) 9 (3.4%)	138 (83.6%) 22 (13.3%) 5 (3%)	35 (74.5%) 10 (21.3%) 2 (4.3%)
Number of 600 mg infusions per patient (median, range)	5 (1–11)	5 (1–11)	7 (1–11)	7 (1–11)
Days since prior infusion (median, range)	189 (140–793)	185 (140–198)	217 (199–288)	349 (293–793)
Percentage of infusions with IRR (%)	355 (24.7%)	268 (24.9%)	74 (24.2%)	13 (23.2%)
Percentage of infusions with IRR at prior infusion (%)	472 (32.8%)	368 (34.2%)	89 (29.1%)	15 (26.8%)
Percentage of infusions with IRR per infusion number (%): 1^st^ 600mg 2^nd^ 600mg 3^rd^ 600mg 4^th^ 600mg 5^th^ 600mg 6^th^ 600mg 7^th^ 600mg 8^th^ 600mg 9^th^ 600mg 10^th^ 600mg 11^th^ 600mg	114 / 275 (41.5%) 80 / 248 (32.3%) 56 / 216 (25.9%) 28 / 178 (15.7%) 26 / 142 (18.3%) 24 / 123 (19.5%) 13 / 100 (13%) 8 / 78 (10.2%) 3 / 50 (6%) 3 / 25 (12%) 0 / 3 (0%)	95 / 231 (41.1%) 62 / 182 (34.1%) 36 / 144 (25%) 15 / 108 (13.9%) 20 / 105 (19%) 20 / 98 (20.4%) 8 / 80 (10%) 8 / 64 (12.5%) 2 / 40 (5%) 2 / 21 (9.5%) 0 / 3 (0%)	17 / 37 (45.9%) 14 / 55 (25.5%) 17 / 58 (29.3%) 12 / 61 (19.7%) 5 / 30 (16.7%) 2 / 21 (9.5%) 5 / 19 (26.3%) 0 / 13 (0%) 1 / 9 (11.1%) 1 / 3 (33.3%) -	2 / 7 (28.6%) 4 / 11 (36.4%) 3 / 14 (21.4%) 1 / 9 (11.1%) 1 / 7 (14.3%) 2 / 4 (50%) 0 / 1 (0%) 0 / 1 (0%) 0 / 1 (0%) 0 / 1 (0%) -
Type of premedication Clemastine Levocetirizine None (patients pref.)	896 (62.3%) 538 (37.4%) 4 (0.3%)	665 (61.8%) 408 (37.9%) 3 (0.3%)	193 (63.1%) 112 (36.6%) 1 (0.3%)	38 (67.9%) 18 (32.1%) -
Infusion rate 3.5 h 2 h unknown	681 (47.3%) 632 (43.9%) 125 (8.7%)	541 (50.3%) 445 (41.4%) 90 (8.4%)	117 (38.2%) 162 (52.9%) 27 (8.8%)	23 (41.1%) 25 (44.6%) 8 (14.3%)
CD19+ B-cell count (x10^9^/L) (median, range)	0.002 (0–0.411)	0.002 (0–0.327)	0.011 (0–0.411)	0.02 (0–0.403)

A total of 1158 IRRs were recorded, all of which were mild (92.5%) or moderate (7.5%) in severity on the CTCAE scale. No life-threatening or severe IRRs occurred. The most common mild IRRs were fatigue (21.8%), throat irritation (17.8%), headache (7.7%), pallor (6.5%), and hypotension (5.8%). The most common moderate IRRs were fatigue (40.2%), headache (11.5%), hypertension (8%), hypotension (5.7%), and throat irritation (4.6%). Most IRRs were observed at the first and second 300 mg infusions; 66.5% of patients experienced an IRR during the first 300 mg infusion, and 43.3% during the second. IRR occurrence declined with each subsequent infusion (see Table 1).

Of the 1438 infusions, 306 were given in the moderate (7–9 months) interval and 56 in the long (>9 months) interval. Neither the moderate interval group (OR = 0.914, 95% CI: 0.498–1.674, *p* = 0.77) nor the long interval group (OR = 0.928, 95% CI: 0.715–1.204, *p* = 0.575) had significantly higher odds of developing IRR compared to the normal interval group.

More overall infusions reduced IRR risk (OR = 0.864, 95% CI: 0.795–0.938, *p* < 0.001) while prior IRRs increased the IRR risk (OR = 1.787, 95% CI: 1.337–2.389, *p* < 0.001). A faster infusion rate (2 vs 3.5 h) lowered the odds of having IRR (OR = 0.462, 95% CI: 0.316–0.675, *p* < 0.001), as did premedication with levocetirizine versus clemastine (OR = 0.668, 95% CI: 0.485–0.921, *p* = 0.014). A higher B-cell count at the moment of OCR infusion slightly increased IRR risk (OR = 1.004, 95% CI: 1.000–1.008, *p* = 0.035).

Reasons for the extended interruption varied. In the long-interval group, B-cell tailored dosing was the most common reason (37 patients, 66.1%), followed by pregnancy (12 patients, 21.4%), infection (3 patients, 5.4%), and other reasons (4 patients, 7.1%), including scheduling conflicts, holidays, or fear of infections. This group included seven infusions with patients without a prior history of IRRs in any previous infusion, nor did they develop IRRs during the infusion after an extended interval. Therefore, no infusion that had never previously resulted in an IRR led to a new IRR at the extended infusion. In the long-interval group, CD19+ B-cell counts ranged from 0 to 0.403 × 10^9^/L (median: 0.02 × 10^9^/L).

A total of 43 infusions were administered when CD19+ B-cell counts exceeded the extended dosing threshold (0.01 × 10^9^/L), with IRRs occurring in 11 of these cases (25.6%). Additionally, 5 infusions were given when CD19+ B-cell counts were above the lower limit of normal (LLN: 0.1 × 10^9^/L), and IRRs occurred in two of these cases (40%).

## Discussion

In this retrospective cohort study, extended intervals between infusions (whether moderate or long) did not significantly increase the odds of developing IRRs compared to the standard six-month dosing schedule.

While previous studies have suggested that B-cell repopulation may influence the likelihood of IRRs,^7,8^ our findings indicate that duration of treatment and prior occurrence of IRRs may be more predictive. This further supports the safety and practicality of extended dosing, which results in higher levels of B-cells but does not lead to increased clinical or radiological disease activity.^9–11^

Our analysis suggests that a faster administration schedule appears protective against IRRs. However, this effect is driven by the administration protocol, as only patients without prior IRRs qualified for a higher infusion rate, creating a naturally lower-risk group. Previous studies have shown that shorter OCR infusion times are generally well tolerated and do not lead to an increased risk of IRRs, provided that patients did not experience serious IRRs during earlier infusions.^2,12^ The same holds for levocetirizine, as our data indicate that it is more effective in preventing IRRs than clemastine. However, this effect is largely due to the fact that more patients received levocetirizine at later infusions as part of a general protocol change, and the infusion number likely plays a greater role, rather than the premedication itself. Previous studies investigating different pre-treatment regimens have found that the choice of antihistamine is associated with similar patient outcomes.^13,14^

Overall, it is reassuring that, consistent with previous studies, all the IRRs observed were mild (92.5%) or moderate (7.5%) in severity, with fatigue, throat irritation, headache, hyper-/hypotension and pallor being the most common symptoms.^1,7,12^ The higher incidence of IRRs in our cohort compared to the pivotal trials,^15^ especially at the first and second 300 mg infusions, may be explained by the retrospective nature of our study, as we classified any symptom reported by patients that could potentially be an IRR, including mild symptoms such as fatigue, as an IRR. This approach likely led to a broader definition of IRRs.

Limitations of this study include the retrospective design, the heterogeneity in both premedication types and infusion rates and smaller sample sizes in the extended interval groups, especially in the long interval group (>9 months, n = 56 infusions).

Despite these limitations, our findings indicate that administering OCR as a single 600 mg infusion after a prolonged treatment interval is safe regarding IRR occurrence.
